# Patient acceptance of video consultations in cardiology

**DOI:** 10.1093/ehjdh/ztaf089

**Published:** 2025-09-26

**Authors:** Julia Lortz, Tienush Rassaf, Laura Johannsen, Wibke Tonscheidt, Finley Sam Mellis, Lisa Maria Jahre, Marc Hesenius, Marvin Bachert, Christos Rammos, Martin Teufel, Alexander Bäuerle

**Affiliations:** Department of Cardiology and Vascular Medicine, West-German Heart and Vascular Center Essen, University of Duisburg-Essen, Hufelandstr. 55, Essen 45147, Germany; Department of Cardiology and Vascular Medicine, West-German Heart and Vascular Center Essen, University of Duisburg-Essen, Hufelandstr. 55, Essen 45147, Germany; Department of Cardiology and Vascular Medicine, West-German Heart and Vascular Center Essen, University of Duisburg-Essen, Hufelandstr. 55, Essen 45147, Germany; Department of Cardiology and Vascular Medicine, West-German Heart and Vascular Center Essen, University of Duisburg-Essen, Hufelandstr. 55, Essen 45147, Germany; Clinic for Psychosomatic Medicine and Psychotherapy, LVR-University Hospital Essen, University of Duisburg-Essen, Virchowstr., 174, Essen 45147, Germany; Center for Translational Neuro- and Behavioral Sciences (C-TNBS), University of Duisburg-Essen, Essen, Germany; Clinic for Psychosomatic Medicine and Psychotherapy, LVR-University Hospital Essen, University of Duisburg-Essen, Virchowstr., 174, Essen 45147, Germany; Center for Translational Neuro- and Behavioral Sciences (C-TNBS), University of Duisburg-Essen, Essen, Germany; Institute for Software Engineering, University of Duisburg-Essen, Essen, Germany; Institute for Software Engineering, University of Duisburg-Essen, Essen, Germany; Department of Cardiology and Vascular Medicine, West-German Heart and Vascular Center Essen, University of Duisburg-Essen, Hufelandstr. 55, Essen 45147, Germany; Clinic for Psychosomatic Medicine and Psychotherapy, LVR-University Hospital Essen, University of Duisburg-Essen, Virchowstr., 174, Essen 45147, Germany; Center for Translational Neuro- and Behavioral Sciences (C-TNBS), University of Duisburg-Essen, Essen, Germany; Clinic for Psychosomatic Medicine and Psychotherapy, LVR-University Hospital Essen, University of Duisburg-Essen, Virchowstr., 174, Essen 45147, Germany; Center for Translational Neuro- and Behavioral Sciences (C-TNBS), University of Duisburg-Essen, Essen, Germany

**Keywords:** Telemedicine, Digital health, eHealth, Remote, Digitalization, UTAUT

## Abstract

**Aims:**

Cardiovascular disease is the leading global cause of mortality. Traditional face-to-face cardiovascular care, while effective, poses challenges such as travel burdens and accessibility issues. Video consultations offer a modern solution, improving access and efficiency while reducing patient strain. This study investigates patient acceptance of video consultations in cardiovascular care using a survey-based approach, assessing key factors influencing their integration into routine practice.

**Methods and results:**

A cross-sectional study including patients attending a cardiological university hospital was conducted. Acceptance of video consultations and its associated factors were assessed using a modified assessment instrument based on the unified theory of acceptance and use of technology. The study comprised 337 participants (*M* = 61.13 years, SD = 14.54), 54.6% male. Acceptance was moderate (*M* = 2.88, SD = 1.37), with 30.27% of the participants reporting high acceptance, 28.19% reporting moderate acceptance, and 41.54% low acceptance. Only 3% had used video consultations before. eHealth literacy was high, while digital confidence was moderate. Analysis showed that higher education, digital confidence, and eHealth literacy predicted greater acceptance of video consultations. Effort expectancy, performance expectancy (PE), and social influence (SI) accounted for most of the variance in acceptance (*R*^2^ = 0.724).

**Conclusion:**

We identified moderate acceptance of video consultations in cardiology, with education, digital confidence, eHealth literacy, and PE as key factors associated with acceptance. Despite low prior use, perceived ease of use and SI were most strongly associated with acceptance. Addressing technical concerns and promoting digital literacy may enhance adoption, improving access to remote cardiac care.

## Graphical Abstract

**Graphical Abstract:**
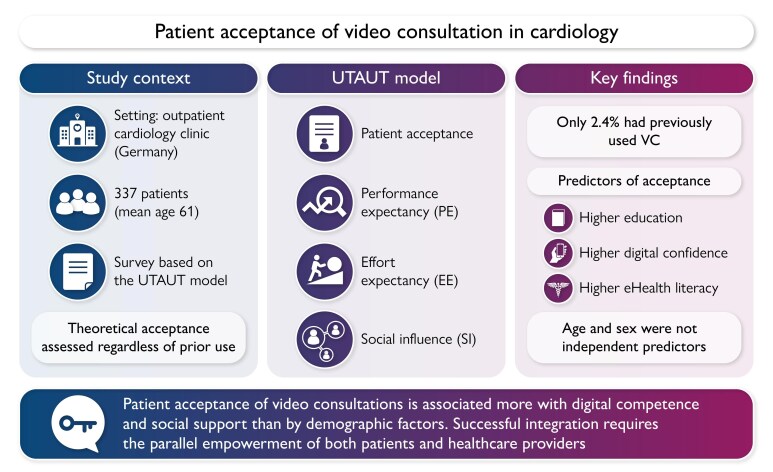

## Introduction

Cardiovascular disease (CVD) is the leading cause of mortality worldwide, accounting for approximately 20.1 million global deaths in 2021.^1^ This burden is set to increase, driven by aging populations, lifestyle factors, and the rise in comorbid conditions such as diabetes and obesity.^1,2^ As a result, CVD continues to pose significant challenges to healthcare systems worldwide.

In the traditional model of cardiovascular care, there is considerable reliance on face-to-face consultations and on hospital-based interventions. This model of care ensures that patients receive personalized and immediate attention from their doctors, fostering trust, and communication. In-person consultations allow for comprehensive physical examinations. Additionally, the traditional model facilitates multi-disciplinary collaboration within the hospital setting, enhancing the quality and optimal coordination of patient care.^3^

This type of medical care has some disadvantages and opens doors to other, modern models of medical care. In-person consultations in hospitals can be time-consuming for patients, often requiring travel and long waiting times, which can be particularly burdensome for those with mobility issues or living in remote areas and lead to high physical stress levels.^4,5^

Telemedicine and video consultations in particular, tries to overcome this disadvantage, allowing for remote consultations and continuity of care. Governments around the world and organizations such as the World Health Organization (WHO) increasingly address the issue of digital medicine, partly as a result of the COVID-19 pandemic, and increasingly engage with telemedicine in their laws and statutes.^6,7^ Digital approaches in medicine, including video consultations, offer promising opportunities by improving access to healthcare services, reducing travel requirements, and minimizing waiting times.^8,9^ While encouraging studies suggest potential benefits, particularly in supporting ongoing disease management, the overall evidence base remains evolving,^10,11^ and further research is needed.

Although the term secondary prevention traditionally refers to the early detection of asymptomatic diseases to enable timely intervention, as seen in screening programmes like mammography, in the context of cardiovascular disease the term is used more broadly to describe efforts aimed at preventing disease progression and complications in patients with existing conditions. In the case of digital health solutions, including telemedicine, their role is often in the ongoing management of chronic diseases, medication adherence, and lifestyle interventions rather than in early disease detection.

Therefore, remote care can enhance monitoring and management of chronic conditions through continuous data collection and real-time communication with healthcare providers. This approach also helps to alleviate the strain on hospital resources, contributing to a more efficient and accessible healthcare system.^12,13^ Video consultations leverage information and communication technologies to provide healthcare services across distances, addressing geographical barriers and improving accessibility.^4,14,15^ However, while these digital solutions offer promising benefits, challenges remain. Issues such as the digital divide, unstable internet connections, difficulties faced by older adults or those with analogue preferences, and varying levels of technological preparedness among healthcare providers have been highlighted in the literature.^16,17^ A balanced approach is necessary to ensure that video consultations complement, rather than replace, traditional healthcare services in an inclusive and effective manner.

Specifically, the use of medical video consultations has become increasingly established in various areas of healthcare.^18–22^ In cardiovascular care they can serve as a valuable complement to in-person visits, particularly for follow-ups, medication management, lifestyle counselling, and psychosocial support. They enable remote monitoring of chronic conditions, might reduce the need for unnecessary hospital visits, and improve accessibility for patients with mobility or geographic barriers. While physical examinations remain essential, video consultations facilitate prescription refills, triage assessments, and integration with remote monitoring technologies, ensuring continuity of care while alleviating strain on healthcare systems.^23^

However, the use and acceptance of video consultations vary considerably across regions and healthcare systems. In countries such as Australia, Canada, and the United States—where large rural areas and long travel distances are common—telemedicine has become more firmly integrated into routine care.^24,25^ In contrast, Germany has historically lagged in digital health adoption. Despite recent efforts such as the Digital Healthcare Act (DVG), the integration of video consultations into standard practice remains limited.^26^ National surveys suggest low awareness and utilization among both patients and providers, though the COVID-19 pandemic has contributed to a gradual shift.^27,28^ Given these disparities, it is important to interpret findings on video consultation acceptance within the context of specific national healthcare environments.

### Objectives

Patients’ acceptance is crucial when introducing new treatment approaches. In Germany, there is limited research on how patients perceive and accept video consultations in the field of cardiology. This study aimed to assess patient acceptance of video consultation in cardiology. Furthermore, its aimed to identify factors associated with the acceptance of video consultations in CVD by examining a diverse set of variables, including sociodemographic characteristics, medical data, eHealth-related factors, and constructs from the well-established unified theory of acceptance and use of technology (UTAUT) model,^29,30^ which aims to explain likelihood of adopting technology with behavioural usage intention, including effort expectancy (EE), performance expectancy (PE), and social influence (SI). The UTAUT model has previously demonstrated its efficacy in explaining people’s willingness to use technology in healthcare.^31,32^ The goal was to provide insights that could guide strategies for improving the adoption and integration of CVD in patient care.

## Methods

### Study design, participants, and procedure

A survey-based, cross-sectional study was conducted to assess acceptance and the factors associated with video consultations for patients attending specialist outpatient treatment for cardiac and related diseases. Participants were recruited in person between March 2024 and August 2024 at the Department of Cardiology and Vascular Medicine, West German Heart and Vascular Center, University Hospital Essen. Recruitment materials included information on study conductors, purpose of the study, anonymity of participation, and an estimate about the time the study would take to complete. A QR code providing access to the survey was given to eligible patients once in the outpatient department in the waiting room, without any follow-up or reminders. The survey was conducted at a single point in time and participants completed it at their convenience, either at home or in a private setting. The researchers were not present during the data collection process to ensure privacy and reduce any potential bias. Participation was voluntary, and responses were submitted electronically, eliminating the need for paper-based submissions. The survey was implemented on the platform Unipark and was accessible from March 2024 to August 2024. All participants electronically provided their informed consent prior to the start of the assessment. Participation in the study was voluntary, anonymous, and with no monetary compensation. Participants who attended a specialist outpatient treatment for cardiac and related diseases with a legal age of 18 years or older, sufficient skills of the German language and Internet access were eligible to participate. The assessment was developed by experts in the field (cardiologists, eHealth-experts, and psychologists). Prior to the start of the study, two individuals without involvement in the study tested the assessment regarding its functionality and clarity of the content. The items were displayed in their validated order on six pages with a maximum of 32 items per page. As the complexity of the questionnaire was deliberately kept low, adaptive questioning was not necessary. Participants did not have the option to review or change answers. Answering applicable items was mandatory to complete the survey. Participants’ processing time was checked for unusually long or short times, but none were excluded.

To uphold high methodological standards, the checklist for reporting results of internet E-surveys^33^ and the strengthening the reporting of observational studies in epidemiology^34^ guidelines were followed (see Supplementary material online, Appendix S1 and Tables A1 and A2). The Ethics Committee of the Medical Faculty of the University of Duisburg–Essen approved the conductance of the study (19-89-47-BO).

Initially, a sample of *N* = 364 started processing the assessment, of which *n* = 332 completed it, representing a completion rate of 91.21%. Of all participants, *n* = 10 (2.75%) were excluded due to not meeting the inclusion criteria. Further, *n* = 17 (4.67%) participants were excluded because of missing values on the primary outcome (acceptance). Therefore, *N* = 337 (92.58%) participants were included in the final data analysis. The average completion time was *M* = 6:32 min (SD = 7:47 min).

#### Assessment instruments

Sociodemographic (section one, *n* = 10), medical (section two, *n* = 24), and eHealth-related data (section three, *n* = 42) were assessed. The acceptance of video consultations among cardiac patients and its drivers and barriers were determined by using a modified version of the UTAUT model (section four, *n* = 14). The assessment is provided in the appendix (see Supplementary material online, Appendix  *S2*).

### Sociodemographic and medical data

The sociodemographic data gathered were age (years), sex (male, female, divers), marital status (married, living in a partnership, single, divorced/separated, widowed, and other), level of education (left school without qualification, certificate of primary/secondary education, general certificate of secondary education, A-levels or entrance qualification for universities, university degree, academic degree, and other), occupational status (still in education, not employed, on sick leave, part-time employment, full-time employment, and retired/pensioned), fitness for work (yes, no), place of residence (large city, medium-sized, small town, and rural community), and living situation (alone, with a partner, in a residential facility). Additionally, participants were asked whether they have a care grade (no, care degrees from 1 to 5, unknown) and what distance they had to travel to their cardiologist (km).

Regarding medical data, participants were asked if they had a cardiac or vascular disease, which was specified in the following questions (Congestive heart failure, myocardial infarction, and cardiac arrhythmia), also in terms of current symptoms related to their CVD. They also stated if they had undergone bypass, pacemaker, or defibrillator implantation surgery. They were asked about the occurrence of the following symptoms: breathlessness at rest and under stress, palpitation, oedema, vertigo, and syncope. Stamina was assessed by asking both how many stairs they could take (0–4, no restrictions) and how long they could walk without experiencing shortness of breath or a feeling of constriction in their chest (0–5 min, 5–10 min, 10–20 min, 20–30 min, 30–40 min, >40 min, no restrictions). Additionally, their past and present smoking habits were assessed. Further, participants rated their subjective physical health on a range from 0 (‘very bad health’) to 10 (‘very good health’).

Regarding mental health data, participants were asked whether they were diagnosed with a mental illness. To assess depressive symptoms over the last two weeks, the Patient Health Questionnaire Scale-8 (PHQ-8^35^) was used. The scale consists of eight items and responses are given on a four-point Likert scale (0 = ‘never’ to 4 = ‘nearly every day’). A cut-off score of ≥ 10 indicates current major depressive symptoms. Internal consistency was high (Cronbach’s α = 0.87). Participants rated their subjective mental health on a range from 0 (‘very bad health’) to 10 (‘very good health’). They further rated their current quality of life on a range from 0 (‘very bad quality of life’) to 10 (‘very good quality of life’). Current distress was assessed with the visual scale of the distress thermometer,^36^ which ranges from 0 (‘no distress’) to 10 (‘extreme distress’). Elevated distress is indicated by a score of ≥4 points.

### eHealth-related data

eHealth literacy, meaning the participant’s ability to gather and understand health information from electronic sources, was assessed using the revised German version of the eHealth Literacy Scale (GR-eHEALS;^37,38^), with responses given on a five-point Likert scale (1 = ‘strongly disagree’ to 5 = ‘strongly agree’). Its internal consistency was excellent (Cronbach’s α = 0.97). Additionally, participants reported their knowledge about digital cardiological health promotion, with responses to three items given on a five-point Likert scale (1 = ‘strongly disagree’ to 5 = ‘strongly agree’). Internal consistency was excellent (Cronbach’s α = 0.92). These items were adapted from a previous study.^39^ Participants also assessed their digital confidence by rating four items on a five-point Likert scale (1 = ‘very insecure’ to 5 = ‘very confident’).^40^ Internal consistency was excellent (Cronbach’s α = 0.95). Further, digital overload was assessed with three items, with answers given on a five-point Likert scale (1 = ‘does not apply’ to 5 = ‘does fully apply’).^32^ The internal consistency was high (Cronbach’s α = 0.85). Internet anxiety was assessed using three items. Answers were given on a five-point Likert scale (1 = ‘does not apply’ to 5 = ‘does fully apply’).^41^ The internal consistency was high (Cronbach’s α = 0.88). Participants were asked whether they were aware of the possibility of communicating with a doctor via video consultation, whether their treating cardiologist offered video consultation, and whether they had used cardiology video consultation in the past (more than once, once, no). They stated for what kinds of problems they would potentially use video consultation (minor medical problems, questions about medication, questions about lifestyle changes, follow-up, prescriptions/certificates of incapacity for work, discussion of findings/examination of results, and first consultation), and what kinds of digital services they had already used in health care (online pharmacy, online health portals/website, mobile health, online support services, electronic patient file, online appointment booking, online orders of prescriptions, telemedicine, and none). Additionally, they stated what they considered to be main reasons for not using video consultation in cardiology (preference of personal contact, lack of technical requirements, fear of incorrect remote diagnostics, and lack of data protection). Last, participants reported the importance of personal trust with their cardiologist prior to the use of video consultations (one item, adapted from^41^) on a five-point Likert scale (1 = ‘very important’ to 5 = ‘not important’).

### UTAUT variables and acceptance

To assess the acceptance of video consultations and its associated factors, a modified version of the UTAUT questionnaire,^29,30,42^ adapted to the research objectives was used. Three independent researchers adapted the UTAUT items to the research objective. First, recent literature was assessed by these researchers to generate items that match to items used in recently published articles. Based on the literature assessment, the UTAUT items were adjusted for video consultations in the field of cardiology. An interdisciplinary panel of psychologists, experts in digital health and senior physicians, specialized in cardiology, evaluated and refined the items through multiple feedback rounds. Through two iterative feedback rounds, input was collected, revisions were implemented accordingly, and the revised version was re-evaluated until a final consensus was reached. The modified UTAUT questionnaire contained 14 items—three assessing acceptance, operationalized as behavioural intention (BI) (e.g. ‘I’d like to try the cardiological video consultation’), three assessing SI (e.g. ‘People close to me would approve of using the cardiological video consultation’), five assessing PE (e.g. ‘The cardiological video consultation could improve my general well-being’), and three assessing EE (e.g. ‘Using the cardiological video consultation wouldn’t be an additional burden for me’). Answers were given on a five-point Likert scale (1 = ‘strongly disagree’ to 5 = ‘strongly agree’). Internal consistency ranged from high to excellent (BI: Cronbach’s α = 0.94; SI: α = 0.85; PE: α = 0.89; EE: α = 0.91). Assessing the acceptance of video consultation in medical fields using the UTAUT model is a validated option as previously shown.^41,43^

#### Statistical analyses

Statistical analyses were performed using R (v4.4.1) and RStudio (v2024.9.0.375). Mean scores were calculated for digital confidence, internet anxiety, digital overload, and for each UTAUT scale (BI, SI, PE, and EE). Sum scores were calculated for GR-eHEALS. Acceptance was operationalized as behavioural intention and was further categorized in accordance with prior research^39,42^: scores from 1 to 2.34 indicated low acceptance, scores from 2.35 to 3.67 indicated moderate acceptance, and scores from 3.68 to 5 indicated high acceptance. Descriptive statistics were applied for sociodemographic, medical, and eHealth-related data. Multiple hierarchical regression analysis was conducted to examine drivers of and barriers to the acceptance of video consultations among cardiac patients. Variables were included block-wise: (i) sociodemographic data, (ii) medical data, (iii) eHealth-related data, and (iv) UTAUT variables. The generalized variance inflation factor (GVIF) was used to verify the absence of multi-collinearity (all GVIF values < 2.0^44^). A visual inspection of Q–Q plots of the residuals showed no signs of violations against normality. Therefore, the normal distribution of the residuals was assumed. Scatter-plots of the standardized residuals and the adjusted predicted values verified homoscedasticity. The level of significance was set to α < 0.05 for all tests. Effect sizes were reported according to Cohen,^45^ with values around 0.2, 0.5, and 0.8 indicating small, medium, and large effects, respectively.

## Results

### Study population

On average, participants were 61.1 years old (SD = 14.5), with ages ranging from 19 to 93 years. Slightly more than half of the sample identified as male (54.6%). Most participants were well-educated, with over 64% holding at least a secondary school certificate. *Table 1* presents a comprehensive overview of the study population.

**Table 1:** Sample characteristics

	*M* (SD)	*n* (%)
Sex		
Male		184 (54.6)
Female		152 (45.1)
Diverse		1 (0.3)
Age	61.1 (14.5)	
Marital status		
Single		37 (11.0)
In a relationship		29 (8.6)
Married		188 (55.8)
Divorced/separated		48 (14.2)
Widowed		35 (10.4)
Educational level		
No/lower education/other		119 (35.3)
Secondary education		91 (27.0)
University entrance qualification		57 (16.9)
University education		70 (20.8)
Currently employed		149 (44.2)
Currently unfit for work		64 (19.0)
Place of residence		
Large city (>100 000)		240 (71.2)
Medium-sized city (>20 000)		75 (22.3)
Small city or rural area (≤20 000)		22 (6.5)
Living situation		
Alone		98 (29.1)
With a partner		233 (69.1)
In a residential facility		6 (1.8)
Care degree		62 (18.4)
Kind of cardiovascular disease and related symptoms		
Cardiac disease		207 (61.4)
Vascular disease		195 (57.9)
Congestive heart failure		109 (32.3)
Myocardial infarction		48 (14.2)
Coronary artery bypass graft		107 (31.8)
Cardiac arrhythmia		80 (23.7)
Cardiac pacemaker		25 (7.4)
Shortness of breath at rest		25 (7.4)
Shortness of breath under stress		225 (66.8)
Palpitation		86 (25.5)
Oedema		95 (28.2)
Vertigo		98 (29.1)
Syncope		10 (3.0)
Ability to climb stairs without shortness of breath or feeling of constriction in the chest		
1 flight of stairs		16 (4.7)
2 flights of stairs		45 (13.4)
3 flights of stairs		86 (25.5)
4 flights of stairs		29 (8.6)
No restrictions		113 (33.5)
Walking distance without shortness of breath or feeling of constriction in the chest (min)		
0–5		31 (9.2)
5–10		31 (92.)
10–20		45 (13.4)
20–30		44 (13.1)
30–40		14 (4.2)
>40		19 (5.6)
No restrictions		153 (45.4)
Smoking habits		
Current smoker		76 (22.6)
Former smoker		101 (30.0)
Never smoked		160 (47.5)
Diagnosed mental disorder		55 (16.3)
PHQ-8	5.2 (4.8)	
Major depressive symptoms		65 (19.3)
Physical health (range 0–10)	5.3 (2.2)	
Mental health (range 0–10)	6.5 (2.6)	
Quality of life (range 0–10)	6.3 (2.4)	
Distress (range 0–10)	5.1 (2.5)	
Elevated distress		244 (72.4)
eHealth literacy (range 8–40)	25.9 (9.6)	
eHealth knowledge (range 1–5)	2.5 (1.2)	
Digital confidence (range 1–5)	3.3 (1.1)	
Digital overload (range 1–5)	2.1 (1.0)	
Internet anxiety (range 1–5)	1.8 (1.0)	
Total		337 (100.0)

In terms of health-related variables, participants reported moderate levels of physical health (*M* = 5.3, SD = 2.2, range = 0–10), mental health (*M* = 6.5, SD = 2.6), and quality of life (*M* = 6.3, SD = 2.4). Average distress levels were 5.1 (SD = 2.5), and 72.4% (*n* = 244) scored above the threshold for elevated distress (≥4). Regarding depressive symptoms, the mean PHQ-8 score was 5.2 (SD = 4.8), with 19.3% (*n* = 65) reaching the cut-off for major depressive symptoms (≥10).

Participants reported moderate levels of eHealth literacy (*M* = 25.9, SD = 9.6, range = 8–40), aligning with previous studies that indicate no established norms for classification.^46,47^ Given the skewed distribution, eHEALS sum scores were grouped into three categories: low (8–19), moderate (20–29), and high (30–40), to better reflect digital health literacy levels. The majority of participants fell into the moderate or high literacy categories (see *Table 1*). eHealth-related knowledge (*M* = 2.5, SD = 1.2, range = 1–5) and digital confidence (*M* = 3.3, SD = 1.1) were also moderate. Digital overload (*M* = 2.1, SD = 1.0) and Internet anxiety (*M* = 1.8, SD = 1.0) were relatively low.

#### Acceptance of video consultations in cardiology

**Figure 1:**
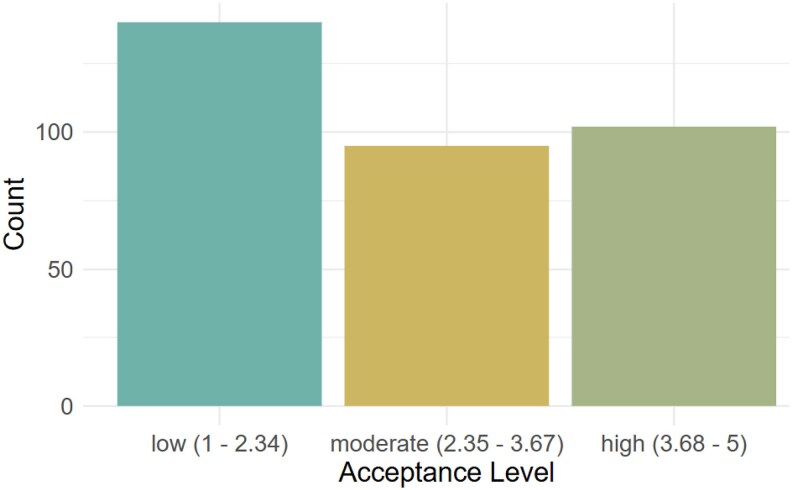
Distribution of acceptance levels among participants. The figure presents both categorical and continuous representations of acceptance, illustrating the range and frequency of responses. Thresholds for low, moderate, and high acceptance were determined based on previous research.

Overall, the acceptance of video consultations among cardiac patients was moderate (*M* = 2.9, SD = 1.4, range 1–5). More precisely, 30.3% (*n* = 102) of participants reported high acceptance, 28.2% (*n* = 95) reported moderate acceptance, and 41.5% (*n* = 140) reported low acceptance (*Figure 1*; *Table 2*).

**Table 2:** Participants responses towards video consultations in cardiology

	*n* (%)
Informed about video consultations	69 (20.5)
Availability of video consultations at cardiologist	
Yes	10 (3.0)
No	123 (36.5)
I don’t know	200 (59.3)
Prior use of video consultations	
More than once	3 (0.9)
Once	5 (1.5)
No	325 (96.4)
Potential use of video consultations for	
Minor medical problems	175 (51.9)
Questions about medication	169 (50.1)
Questions about lifestyle changes	85 (25.2)
Follow-up	107 (31.8)
Prescription/certificate of incapacity for work	130 (38.6)
Discussion of findings/examinations of results	142 (42.1)
First consultation	34 (10.1)
Need of personal trust with the cardiologist prior to the use of video consultations	
Very important	170 (48.0)
Rather important	77 (21.8)
Neutral	53 (15.0)
Rather unimportant	15 (4.2)
Not important at all	18 (5.1)
Barriers of video consultations use	
Preference of personal contact with physician	254 (75.4)
Lack of technical requirements	64 (19.0)
Fear of incorrect remote diagnostics	78 (23.1)
Lack of private data protection	41 (12.2)
Prior use of digital services in health care	
Online pharmacy	125 (37.1)
Online health portals/website	65 (19.3)
Mobile health (e.g. health trackers)	77 (22.8)
Online support services	15 (4.5)
Electronic patient file	27 (8.0)
Online appointment booking	167 (49.6)
Online orders of prescriptions	88 (26.1)
Telemedicine (e.g. video consultations, e-mail)	22 (6.5)
None	119 (35.3)
Missing data	4 (1.2)
Total	337 (100.0)

#### Factors associated with acceptance of video consultations in cardiology

Multiple hierarchical regression analysis was performed to determine factors associated with the acceptance of video consultations among cardiac patients. Due to missing data on the independent variables and to ensure the necessary participant number per category, *n* = 6 participants had to be excluded from the analysis.

In the first step, sociodemographic data were included (*R*^2^ = 0.113, $R_{\mathrm{adj}}^{2}$ = 0.102, *P* < 0.001), explaining 11.3% of variance in acceptance. None of the sociodemographic variables were significantly associated with acceptance.

In the second step, medical data were included (*R*^2^ = 0.135, $R_{\mathrm{adj}}^{2}$ = 0.116, *P* < 0.001), significantly increasing the explained variance to 13.5% (Δ*R*^2^ = 0.022, *F*(7323) = 7.17, *P* < 0.001). None of the medical variables were significantly associated with acceptance.

In the third step, eHealth-related data were included (*R*^2^ = 0.379, $R_{\mathrm{adj}}^{2}$ = 0.357, *P* < 0.001), significantly increasing the explained variance to 37.9% (Δ*R*^2^ = 0.244, *F*(11,319) = 17.66, *P* < 0.001). A significant positive association was found for higher digital confidence (β = 0.15, *P* = 0.001).

In the final step, the three UTAUT variables were included (*R*^2^ = 0.722, $R_{\mathrm{adj}}^{2}$ = 0.709, *P* < 0.001), significantly increasing the explained variance of the final model to 72.2% (Δ*R*^2^ = 0.343, *F*(14,316) = 58.55, *P* < 0.001). Significant factors associated with greater acceptance of video consultations were higher EE (β = 0.36, *P* < 0.001), higher PE (β = 0.16, *P* = 0.002), and greater SI (β = 0.29, *P* < 0.001). *Table 3* shows the final UTAUT model of acceptance and its associated factors.

**Table 3:** Hierarchical regression model of acceptance of video consultations among cardiac Patients

Independent variables	β	SE	*t*	*R* ^2^	Δ*R^2^*	*P*
(Intercept)	0.02	0.06	0.37			0.354
Step 1: Sociodemographic data				0.113	0.113	
Sex (male vs. female = reference)	0.05	0.06	0.77			0.442
Age (in years)	−0.02	0.04	−0.53			0.599
Higher education (yes vs. no = reference)	−0.04	0.07	−0.62			0.538
Employment (employed vs. not = reference)	−0.06	0.08	−0.82			0.412
Step 2: Medical data				0.135	0.022	
Distance to cardiologist (in km)	0.02	0.03	0.69			0.488
PHQ-8 score (0 = no symptoms, 24 = severe symptoms)	0.05	0.04	1.52			0.130
Physical health (1 = poor, 5 = excellent)	0.02	0.03	0.60			0.550
Step 3: eHealth data				0.379	0.244	
Digital overload (1 = low, 5 = high)	−0.00	0.04	−0.00			0.997
Internet anxiety (1 = low, 5 = high)	−0.05	0.04	−1.24			0.215
Digital confidence (1 = low, 5 = high)	0.15	0.05	3.19			0.001
eHealth literacy (8 = low, 40 = high)	0.02	0.04	0.52			0.753
Step 4: UTAUT variables				0.722	0.343	
Effort expectancy (1 = low, 5 = high)	0.36	0.06	6.45			<0.001
Performance expectancy (1 = low, 5 = high)	0.16	0.05	3.06			0.002
Social influence (1 = low, 5 = high)	0.29	0.05	5.75			<0.001

*N* = 331. The dependent variable was acceptance of video consultations, operationalized as behavioural intention on a 5-point Likert scale (1 = low, 5 = high). Independent variables were entered block-wise across four steps. Only newly added variables are shown at each step. PHQ-8, Patient Health Questionnaire-8; β, standardized beta; SE, standard error; *t*, test statistic; *R*^2^, coefficient of determination; Δ*R*^2^, change in *R*^2^.

## Discussion

This study examined the general acceptance of video consultations in cardiology among patients with CVD and identifying associated factors. While previous research has primarily focused on the utilization and feasibility of video consultations in general patient populations and those with CVD, studies investigating factors associated with acceptance remain scarce.^48,49^ Additionally, the acceptance of video consultations has not been extensively analysed through the lens of the UTAUT.

Overall, acceptance of video consultations was moderate among cardiac patients. Only about 30% of participants expressed high acceptance, while over 40% reported low acceptance. Previous studies showed mixed findings in terms of video consultations acceptance indicating that acceptance varies across medical disciplines and patient populations.^50–52^ Similar to findings in oncology and primary care, patients demonstrated a preference for in-person consultations, likely due to the complexity of cardiovascular diseases and the need for physical examinations.^41,53^

The low awareness of video consultations services may also contribute to its limited acceptance, as a majority of participants were unaware of its availability at their cardiologist’s practice. The COVID-19 pandemic has significantly influenced the adoption of telehealth services,^54,55^ but cardiology patients may still require more exposure and education on video consultations benefits to increase its acceptance. It is important to note that although personal experience with video consultations is limited, the UTAUT model measures theoretical acceptance rather than relying solely on direct experience. This means that even participants who have never used video consultations can still assess their acceptance based on perceived usefulness and other relevant factors. The model has been applied in contexts with low actual usage of video consultations, making it a suitable framework for evaluating acceptance in this study.

Although the inclusion of sociodemographic variables in the initial regression steps improved the overall predictive power of the model, none of the individual sociodemographic variables were significantly associated with video consultation acceptance. Subsequent additions of digital competence and UTAUT-related variables at each step led to further significant increases in explained variance, with these constructs emerging as the strongest predictors of acceptance.

Specifically, education and age did not emerge as independent associated factors when psychological and technology-related variables were included. Nonetheless, descriptive data indicated that individuals with higher education levels and younger age tended to report greater acceptance of VCs. This finding aligns with prior research conducted in Germany, which highlights the importance of digital competence over sociodemographic factors in determining eHealth and telemedicine acceptance.^29,41,50^ These studies consistently emphasize that individuals with higher digital literacy and confidence in technology are more likely to accept and use telemedicine services. Sex was not identified as a significant factor in any model. The literature remains inconclusive regarding its role, with some studies reporting higher acceptance among women, while others suggest greater use by men.^43,56^

Medical factors had a limited impact on predicting the acceptance of video consultations. While they slightly increased the explained variance to 14.8%, they were not a significant predictor of telehealth uptake, contrasting with previous findings in oncology.^41^ However, the significant effect of distance to a cardiologist suggests that patients living farther from healthcare facilities may be more likely to adopt video consultations as a convenient alternative.

eHealth-related variables were key associating factors of video consultations acceptance. Higher digital confidence significantly increased acceptance, as patients who feel more comfortable navigating digital platforms are more likely to engage with remote services. Similarly, eHealth literacy emerged as a strong predictor, reinforcing previous research that highlights the importance of digital skills in adoption of video consultations.^57,58^ In contrast, Internet anxiety and digital overload did not have a significant impact, indicating that most patients did not perceive such topics as major barriers to video consultations use.

The UTAUT model demonstrated high predictive value in explaining video consultations acceptance. EE, PE, and SI were all significantly associated with acceptance, with EE and SI showing the strongest effect. As EE emerged as one strong predictor of acceptance of video consultations among cardiac patients, it highlights the critical role of perceived ease of use in adopting remote services. Being aware of the potentially low prior experience with VC, we specifically chose this model to account for theoretical acceptance rather than actual usage. The UTAUT model does not require personal experience with the technology; rather, it assesses acceptance based on factors such as perceived usefulness and ease of use.^42,59^ This allows even non-users to evaluate their acceptance of VC, making the model a suitable framework for our study. Patients who found video consultations easy to use were significantly more likely to accept them, aligning with prior research emphasizing usability as a key determinant of digital health adoption.^60^ The strong association with EE suggests that reducing technological barriers, improving user-friendly interfaces, and providing adequate technical support could enhance acceptance. Future efforts should focus on simplifying digital platforms and offering patient training to improve EE, ultimately increasing the uptake of eHealth services, e.g. the utilization of video consultations in cardiology. SI played an important role in predicting acceptance in this context. This suggests that recommendations from healthcare providers, family, or peers play a crucial role in shaping patient attitudes towards video consultations. The importance of SI aligns with previous findings, where trust in medical professionals and endorsement from caregivers increased acceptance related to video consultations.^61^ PE, reflecting patients’ perceived benefits of video consultations, was also associated with acceptance, indicating that clear communication of video consultations advantages (e.g. convenience and time savings) could enhance uptake.^61^

To increase the adoption of video consultations in cardiology, targeted interventions should address barriers related to age, education, and awareness. Older patients and those with lower education levels may benefit from structured digital literacy trainings and step-by-step guidance on how to use video consultations and in general eHealth services. Healthcare providers should proactively inform patients about the availability of video consultations and emphasize its benefits to improve PE. Additionally, given the strong association with SI, cardiologists and healthcare institutions should actively recommend video consultations to their patients to enhance acceptance.

To address concerns about usability and accessibility, efforts should be made to simplify the video consultation process. Providing technical support, easy-to-use platforms, and alternative options (e.g. phone consultations) may help accommodate patients with lower digital confidence. Future research should explore specific strategies to integrate video consultations into routine cardiac care while ensuring accessibility for all patient demographics.

While this study focused on patient acceptance, the broader context of digitalization in German cardiology practices and the role of healthcare providers must also be considered. Despite progress in digital health, telemedicine remains inconsistently implemented in cardiology, especially in outpatient care. Key barriers include heterogeneous digital infrastructure, lack of training, and concerns about data security—challenges repeatedly noted in the German healthcare system.^27,28^

Importantly, cardiologists’ attitudes significantly influence patient acceptance. Surveys in Germany show that while many physicians recognize the potential of video consultations, hesitations persist due to increased workload, limited reimbursement, and diagnostic concerns without physical examination.^62^ These provider-side reservations may reinforce patient scepticism, particularly given the strong role of SI found in our study. Beyond technology, digital adoption is also a cultural issue. To facilitate broader uptake, cardiologists need greater institutional support, including training programmes, standardized procedures, and integration of video consultations into routine workflows and reimbursement structures.^63^ Future efforts should therefore focus not only on improving patient readiness but also on enabling providers through infrastructure, incentives, and practical guidance, to ensure sustainable implementation of telemedicine in cardiology care.

### Limitations

Some limitations must be acknowledged. Due to the study’s anonymous and open-access design, an accurate participation rate could not be determined. Furthermore, the online format may have introduced sampling bias, potentially under-representing individuals with limited internet access or lower digital literacy. Moreover, the sample consisted of patients visiting a specialized hospital outpatient department, which may not fully represent the broader population using video consultations and limits the generalizability of the reported findings. Further research is needed to compare eHealth literacy with patient groups in primary care and community settings. Moreover, more qualitative methods should be considered to explore patient concerns and preferences in greater depth.

There is an ongoing scientific debate about the best instrument for the assessment of eHealth literacy. In this study, the most established instrument was used. However, there are newer instrument.^64^ Future studies are needed to validate newer and more comprehensive instruments in different languages.

## Conclusions

Video consultations show potential as a supplementary tool in cardiology, but acceptance remains moderate, associated with factors such as educational level and digital confidence. As the study population was drawn from a specialized hospital outpatient department, findings may not fully reflect routine cardiology care. Acceptance levels could differ in primary care or general outpatient settings, highlighting the need for further research. Addressing barriers like limited awareness and low eHealth literacy is essential for broader integration.

## Supplementary Material

ztaf089_Supplementary_Data

## Data Availability

Anonymized data available upon reasonable request.
