# Supplementary material for: Patient acceptance of video consultations in cardiology
Source: Eur Heart J Digit Health. 2025 Sep 26;6(6):1273–81. doi: 10.1093/ehjdh/ztaf089 (PMC12629656; doi:10.1093/ehjdh/ztaf089)
Supplement: ztaf089_Supplementary_Data [file ztaf089_supplementary_data.zip › Appendix 1.docx]

**Patient Acceptance of Video Consultations in Cardiology**

Julia Lortz^1*^, Tienush Rassaf ^1^, Laura Johannsen^1^, Wibke Tonscheidt^1^, Finley Sam Mellis^2,3^, Lisa Maria Jahre^2,3^, Marc Hesenius^4^, Christos Rammos^1^, Martin Teufel^2,3^, Alexander Bäuerle^2,3^

^1^ Department of Cardiology and Vascular Medicine, West-German Heart and Vascular Center Essen, University of Duisburg-Essen, Hufelandstr. 55, 45147 Essen, Germany

^2^ Clinic for Psychosomatic Medicine and Psychotherapy, LVR-University Hospital Essen, University of Duisburg-Essen, Virchowstr., 174, 45147 Essen, Germany

^3^ Center for Translational Neuro- and Behavioral Sciences (C-TNBS), University of Duisburg-Essen, Essen, Germany

^4^ Institute for Software Engineering, University of Duisburg-Essen, Essen, Germany

**Appendix 1**

**Table A1**

*Checklist for Reporting Results of Internet E-Surveys (CHERRIES)*

| **Checklist Item** | **Explanation** | **Section** |
| --- | --- | --- |
| **Design** | | |
| Describe survey design | Describe target population, sample frame. Is the sample a convenience sample? (In “open” surveys this is most likely.) | Methods > Study Design, Participants, and Procedure |
| **IRB (Institutional Review Board) approval and informed consent process** | | |
| IRB approval | Mention whether the study has been approved by an IRB. | Methods > Study Design, Participants, and Procedure |
| Informed consent | Describe the informed consent process. Where were the participants told the length of time of the survey, which data were stored and where and for how long, who the investigator was, and the purpose of the study? | Methods > Study Design, Participants, and Procedure |
| Data protection | If any personal information was collected or stored, describe what mechanisms were used to protect unauthorized access. | No personal information was collected. |
| **Development and pre-testing** | | |
| Development and testing | State how the survey was developed, including whether the usability and technical functionality of the electronic questionnaire had been tested before fielding the questionnaire. | Methods > Assessment Instruments |
| **Recruitment process and description of the sample having access to the questionnaire** | | |
| Open survey versus closed survey | An “open survey” is a survey open for each visitor of a site, while a closed survey is only open to a sample which the investigator knows (password-protected survey). | Methods > Study Design, Participants, and Procedure |
| Contact mode | Indicate whether or not the initial contact with the potential participants was made on the Internet. (Investigators may also send out questionnaires by mail and allow for Web-based data entry.) | Methods > Study Design, Participants, and Procedure |
| Advertising the survey | How/where was the survey announced or advertised? Some examples are offline media (newspapers), or online (mailing lists – If yes, which ones?) or banner ads (Where were these banner ads posted and what did they look like?). It is important to know the wording of the announcement as it will heavily influence who chooses to participate. Ideally the survey announcement should be published as an appendix. | Methods > Study Design, Participants, and Procedure |
| **Survey administration** | | |
| Web/E-mail | State the type of e-survey (eg, one posted on a Web site, or one sent out through e-mail). If it is an e-mail survey, were the responses entered manually into a database, or was there an automatic method for capturing responses? | Methods > Study Design, Participants, and Procedure |
| Context | Describe the Web site (for mailing list/newsgroup) in which the survey was posted. What is the Web site about, who is visiting it, what are visitors normally looking for? Discuss to what degree the content of the Web site could pre-select the sample or influence the results. For example, a survey about vaccination on a anti-immunization Web site will have different results from a Web survey conducted on a government Web site | Discussion > Limitations |
| Mandatory/voluntary | Was it a mandatory survey to be filled in by every visitor who wanted to enter the Web site, or was it a voluntary survey? | Not applicable |
| Incentives | Were any incentives offered (eg, monetary, prizes, or non-monetary incentives such as an offer to provide the survey results)? | Methods > Study Design, Participants, and Procedure |
| Time/Date | In what timeframe were the data collected? | Methods > Study Design, Participants, and Procedure |
| Randomization of items or questionnaires | To prevent biases items can be randomized or alternated. | Methods > Assessment Instruments |
| Adaptive questioning | Use adaptive questioning (certain items, or only conditionally displayed based on responses to other items) to reduce number and complexity of the questions. | Methods > Assessment Instruments |
| Number of Items | What was the number of questionnaire items per page? The number of items is an important factor for the completion rate. | Methods > Assessment Instruments |
| Number of screens (pages) | Over how many pages was the questionnaire distributed? The number of items is an important factor for the completion rate. | Methods > Assessment Instruments |
| Completeness check | It is technically possible to do consistency or completeness checks before the questionnaire is submitted. Was this done, and if “yes”, how (usually JAVAScript)? An alternative is to check for completeness after the questionnaire has been submitted (and highlight mandatory items). If this has been done, it should be reported. All items should provide a non-response option such as “not applicable” or “rather not say”, and selection of one response option should be enforced. | Methods > Assessment Instruments |
| Review step | State whether respondents were able to review and change their answers (eg, through a Back button or a Review step which displays a summary of the responses and asks the respondents if they are correct). | Methods > Assessment Instruments |
| **Response rates** | | |
| Unique site visitor | If you provide view rates or participation rates, you need to define how you determined a unique visitor. There are different techniques available, based on IP addresses or cookies or both. | The platform Unipark (Tivian XI GmbH) determines an unique visitor based on cookies. |
| View rate (Ratio of unique survey visitors/unique site visitors) | Requires counting unique visitors to the first page of the survey, divided by the number of unique site visitors (not page views!). It is not unusual to have view rates of less than 0.1 % if the survey is voluntary. | This data is not accessible to the study team. |
| Participation rate (Ratio of unique visitors who agreed to participate/unique first survey page visitors) | Count the unique number of people who filled in the first survey page (or agreed to participate, for example by checking a checkbox), divided by visitors who visit the first page of the survey (or the informed consents page, if present). This can also be called “recruitment” rate. | Initial page hits to our Unipark survey can not be retrieved by the authors. The recruitment rate can therefore not be calculated. The number of people who agreed to participate is reported in the Methods section (> Study Design, Participants, and Procedure). |
| Completion rate (Ratio of users who finished the survey/users who agreed to participate) | The number of people submitting the last questionnaire page, divided by the number of people who agreed to participate (or submitted the first survey page). This is only relevant if there is a separate “informed consent” page or if the survey goes over several pages. This is a measure for attrition. Note that “completion” can involve leaving questionnaire items blank. This is not a measure for how completely questionnaires were filled in. (If you need a measure for this, use the word “completeness rate”.) | Methods > Assessment Instruments |
| **Preventing multiple entries from the same individual** | | |
| Cookies used | Indicate whether cookies were used to assign a unique user identifier to each client computer. If so, mention the page on which the cookie was set and read, and how long the cookie was valid. Were duplicate entries avoided by preventing users access to the survey twice; or were duplicate database entries having the same user ID eliminated before analysis? In the latter case, which entries were kept for analysis (eg, the first entry or the most recent)? | Unique users were identified via cookies. Duplicate entries were avoided by preventing users access to the survey twice. |
| IP check | Indicate whether the IP address of the client computer was used to identify potential duplicate entries from the same user. If so, mention the period of time for which no two entries from the same IP address were allowed (eg, 24 hours). Were duplicate entries avoided by preventing users with the same IP address access to the survey twice; or were duplicate database entries having the same IP address within a given period of time eliminated before analysis? If the latter, which entries were kept for analysis (eg, the first entry or the most recent)? | IP adresses of users of the platform Unipark (Tivian XI GmbH) are not accessible to the authors. |
| Log file analysis | Indicate whether other techniques to analyze the log file for identification of multiple entries were used. If so, please describe. | No log file analysis was conducted. |
| Registration | In “closed” (non-open) surveys, users need to login first and it is easier to prevent duplicate entries from the same user. Describe how this was done. For example, was the survey never displayed a second time once the user had filled it in, or was the username stored together with the survey results and later eliminated? If the latter, which entries were kept for analysis (eg, the first entry or the most recent)? | Not applicable (open survey). |
| **Analysis** | | |
| Handling of incomplete questionnaires | Were only completed questionnaires analyzed? Were questionnaires which terminated early (where, for example, users did not go through all questionnaire pages) also analyzed? | Methods > Study Design, Participants, and Procedure |
| Questionnaires submitted with an atypical timestamp | Some investigators may measure the time people needed to fill in a questionnaire and exclude questionnaires that were submitted too soon. Specify the timeframe that was used as a cut-off point, and describe how this point was determined. | Methods > Assessment Instruments |
| Statistical correction | Indicate whether any methods such as weighting of items or propensity scores have been used to adjust for the non-representative sample; if so, please describe the methods. | Methods > Statistical Analyses |

**Table A2**

*The Strengthening the Reporting of Observational Studies in Epidemiology (STROBE) Statement: Guidelines for Reporting Observational Studies*

|  | Item No | Recommendation | Section |
| --- | --- | --- | --- |
| **Title and abstract** | 1 | (a) Indicate the study’s design with a commonly used term in the title or the abstract | Title |
|  |  | (b) Provide in the abstract an informative and balanced summary of what was done and what was found | Abstract |
| Introduction | | |  |
| Background/rationale | 2 | Explain the scientific background and rationale for the investigation being reported | Introduction |
| Objectives | 3 | State specific objectives, including any prespecified hypotheses | Introduction |
| Methods | | |  |
| Study design | 4 | Present key elements of study design early in the paper | Methods > Study Design, Participants, and Procedure |
| Setting | 5 | Describe the setting, locations, and relevant dates, including periods of recruitment, exposure, follow-up, and data collection | Methods > Study Design, Participants, and Procedure |
| Participants | 6 | (a) Give the eligibility criteria, and the sources and methods of selection of participants | Methods > Study Design, Participants, and Procedure |
| Variables | 7 | Clearly define all outcomes, exposures, predictors, potential confounders, and effect modifiers. Give diagnostic criteria, if applicable | Methods > Study Design, Participants, and Procedure  Methods > Statistical Analyses |
| Data sources/ measurement | 8* | For each variable of interest, give sources of data and details of methods of assessment (measurement). Describe comparability of assessment methods if there is more than one group | Methods > Assessment Instruments |
| Bias | 9 | Describe any efforts to address potential sources of bias | Methods > Statistical Analysis Limitations |
| Study size | 10 | Explain how the study size was arrived at | Methods > Study Design, Participants, and Procedure |
| Quantitative variables | 11 | Explain how quantitative variables were handled in the analyses. If applicable, describe which groupings were chosen and why | Methods > Statistical Analyses |
| Statistical methods | 12 | (a) Describe all statistical methods, including those used to control for confounding | Methods > Statistical Analyses |
|  |  | (b) Describe any methods used to examine subgroups and interactions | Not applicable. |
|  |  | (c) Explain how missing data were addressed | Methods > Study Design, Participants, and Procedure |
|  |  | (d) If applicable, describe analytical methods taking account of sampling strategy | Not applicable. |
|  |  | (e) Describe any sensitivity analyses | Not applicable. |
| Results | | |  |
| Participants | 13* | (a) Report numbers of individuals at each stage of study—eg numbers potentially eligible, examined for eligibility, confirmed eligible, included in the study, completing follow-up, and analysed | Methods > Study Design, Participants, and Procedure |
|  |  | (b) Give reasons for non-participation at each stage | Not applicable. |
|  |  | (c) Consider use of a flow diagram | Not applicable. |
| Descriptive data | 14* | (a) Give characteristics of study participants (eg demographic, clinical, social) and information on exposures and potential confounders | Methods > Study Design, Participants, and Procedure |
|  |  | (b) Indicate number of participants with missing data for each variable of interest | Methods > Study Design, Participants, and Procedure Results > Table 2 |
| Outcome data | 15* | Report numbers of outcome events or summary measures | Methods > Statistical Analyses |
| Main results | 16 | (a) Give unadjusted estimates and, if applicable, confounder-adjusted estimates and their precision (eg, 95% confidence interval). Make clear which confounders were adjusted for and why they were included | Results > Predictors of Acceptance of Video Consultation in Cardiology  Not applicable |
|  |  | (b) Report category boundaries when continuous variables were categorized | Methods > Statistical Analyses |
|  |  | (c) If relevant, consider translating estimates of relative risk into absolute risk for a meaningful time period | Not applicable. |
| Other analyses | 17 | Report other analyses done—eg analyses of subgroups and interactions, and sensitivity analyses | Not applicable. |
| Discussion | | |  |
| Key results | 18 | Summarise key results with reference to study objectives | Discussion |
| Limitations | 19 | Discuss limitations of the study, taking into account sources of potential bias or imprecision. Discuss both direction and magnitude of any potential bias | Discussion > Limitations |
| Interpretation | 20 | Give a cautious overall interpretation of results considering objectives, limitations, multiplicity of analyses, results from similar studies, and other relevant evidence | Discussion |
| Generalisability | 21 | Discuss the generalisability (external validity) of the study results | Discussion Discussion > Limitations |
| Other information | | |  |
| Funding | 22 | Give the source of funding and the role of the funders for the present study and, if applicable, for the original study on which the present article is based | Funding (Not applicable) |
