# Supplementary material for: Patient acceptance of video consultations in cardiology
Source: Eur Heart J Digit Health. 2025 Sep 26;6(6):1273–81. doi: 10.1093/ehjdh/ztaf089 (PMC12629656; doi:10.1093/ehjdh/ztaf089)
Supplement: ztaf089_Supplementary_Data [file ztaf089_supplementary_data.zip › Appendix 2.docx]

**Patient Acceptance of Video Consultations in Cardiology**

Julia Lortz^1*^, Tienush Rassaf ^1^, Laura Johannsen^1^, Wibke Tonscheidt^1^, Finley Sam Mellis^2,3^, Lisa Maria Jahre^2,3^, Marc Hesenius^4^, Christos Rammos^1^, Martin Teufel^2,3^, Alexander Bäuerle^2,3^

^1^ Department of Cardiology and Vascular Medicine, West-German Heart and Vascular Center Essen, University of Duisburg-Essen, Hufelandstr. 55, 45147 Essen, Germany

^2^ Clinic for Psychosomatic Medicine and Psychotherapy, LVR-University Hospital Essen, University of Duisburg-Essen, Virchowstr., 174, 45147 Essen, Germany

^3^ Center for Translational Neuro- and Behavioral Sciences (C-TNBS), University of Duisburg-Essen, Essen, Germany

^4^ Institute for Software Engineering, University of Duisburg-Essen, Essen, Germany

**Appendix 2**

**Questionnaire: English-Version**

*Original in German. Translated to English for publication purposes only.*

**Sociodemographics**

1. **Please enter your age**:

_________ Years

1. **Please enter your gender**:

| - Male | - Female | - Diverse |
| --- | --- | --- |

1. **Please state your current marital status**

- Married
- Living in a partnership
- Single
- Divorced/separated
- Widowed

1. **What is your highest educational qualification?**

- Left school without qualification
- Certificate of primary/secondary education
- General certificate of secondary education
- A-levels or entrance qualification for universities
- University degree
- Academic degree
- Other

1. **Please state your current occupational status**

- Still in education (e.g. school, university)
- Not employed (e.g. looking for work, unable to work)
- On sick leave
- Part-time employment
- Full-time employment
- Retired/pensioned
- Other

1. **Are you currently unable to work?**

- Yes
- No

1. **Please indicate the type of city or municipality you live in**

- Large city (100,000 residents or more)
- Medium-sized city (20,000 residents or more)
- Small city (5,000 residents or more)
- Rural community (less than 5,000 residents)

1. **What describes your current living situation best?**

- I live alone
- I live with my partner
- I live in a residential facility (retirement home, assisted living)

1. **Do you have a care degree?**

- No, no care degree
- Care degree 1
- Care degree 2
- Care degree 3
- Care degree 4
- Care degree 5
- I don’t know

1. **Please indicate how far away you live from your treating cardiologist.**

*Indicate in kilometers. If you live more than 100km away, please click 100. E.g. distance to a practice or a hospital (use the slider).*

**Cardiological Anamnesis**

1. **Do you have a heart condition (diagnosed by a specialist)?**

| - Yes | - No |
| --- | --- |

1. **Do you have any other vascular diseases (diagnosed by a specialist)?**

| - Yes | - No |
| --- | --- |

1. **Have you been diagnosed with heart failure (diagnosed by a specialist)?**

| - Yes | - No |
| --- | --- |

1. **Have you ever had a heart attack?**

| - Yes | - No |
| --- | --- |

1. **Do you have a heart stent or bypass?**

| - Yes | - No |
| --- | --- |

1. **Do you suffer from cardiac arrhythmia?**

| - Yes | - No |
| --- | --- |

1. **Do you have a pacemaker and/or a defibrillator (defi for short)?**

| - Yes | - No |
| --- | --- |

1. **Which of the following symptoms apply to you?**
   - Shortness of breath at rest
   - Shortness of breath under stress
   - Palpitations
   - Oedema
   - Vertigo
   - Syncope
2. **How many flights of stairs or walking distance can you manage without a break before shortness of breath or a feeling of tightness in the chest occurs?**
   - 0
   - 1
   - 2
   - 3
   - 4
   - No restrictions
3. **How much distance you walk without a break before shortness of breath or a feeling of tightness in the chest occurs?**
   - 0 – 5 minutes
   - 5 – 10 minutes
   - 10 – 20 minutes
   - 20 – 30 minutes
   - 30 – 40 minutes
   - > 40 minutes
   - No restrictions
4. **Do you smoke?**

- Yes
- I used to smoke.
- No

**Psychological Anamnesis**

1. **Do you have or or have been diagnosed with a mental disorder (diagnosed by a doctor or psychotherapist?**

- Yes
- No

1. **How often have you felt affected by the following complaints in the last 2 weeks? Please mark the answer that applies to you.**
   1. **Little interest or pleasure in your activities**
      - Never
      - On some days
      - On more than half of the days
      - Almost every day
   2. **Depression, melancholy or hopelessness**
      - Never
      - On some days
      - On more than half of the days
      - Almost every day
   3. **Difficulty falling/staying asleep or increased sleep**
      - Never
      - On some days
      - On more than half of the days
      - Almost every day
   4. **Tiredness or having no energy**
      - Never
      - On some days
      - On more than half of the days
      - Almost every day
   5. **Decreased appetite or excessive need to eat**
      - Never
      - On some days
      - On more than half of the days
      - Almost every day
   6. **Bad opinion of yourself; feeling like a failure or like having disappointed family**
      - Never
      - On some days
      - On more than half of the days
      - Almost every day
   7. **Difficulty to focus, e.g. reading the newspaper or watching TV**
      - Never
      - On some days
      - On more than half of the days
      - Almost every day
   8. **Were your movements or speech so slowed down that others would notice? Or, on the contrary, were you “fidgety” or restless and therefore had a stronger urge to move than usual?**
      - Never
      - On some days
      - On more than half of the days
      - Almost every day
2. **On a scale from 0 to 10, how would you rate your physical health (e.g. no physical limitations, pain)?**

|  | **0** | **1** | **2** | **3** | **4** | **5** | **6** | **7** | **8** | **9** | **10** |  |
| --- | --- | --- | --- | --- | --- | --- | --- | --- | --- | --- | --- | --- |
| **Very bad health** | **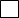** | **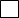** | **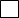** | **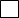** | **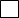** | **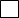** | **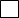** | **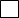** | **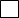** | **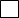** | **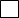** | **Very good health** |

1. **On a scale from 0 to 10, how would you rate your mental health (e.g. no feelings of anxiety, depression)?**

|  | **0** | **1** | **2** | **3** | **4** | **5** | **6** | **7** | **8** | **9** | **10** |  |
| --- | --- | --- | --- | --- | --- | --- | --- | --- | --- | --- | --- | --- |
| **Very bad health** | **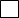** | **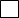** | **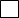** | **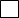** | **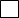** | **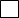** | **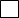** | **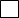** | **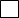** | **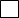** | **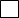** | **Very good health** |

1. **On a scale from 0 to 10, how would you rate your current quality of life?**

|  | **0** | **1** | **2** | **3** | **4** | **5** | **6** | **7** | **8** | **9** | **10** |  |
| --- | --- | --- | --- | --- | --- | --- | --- | --- | --- | --- | --- | --- |
| **Very bad quality of life** | **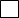** | **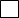** | **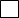** | **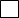** | **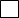** | **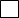** | **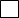** | **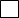** | **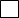** | **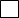** | **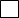** | **Very good quality of life** |

1. **On a scale from 0 to 10, how distressed did you feel last week?**

|  | **0** | **1** | **2** | **3** | **4** | **5** | **6** | **7** | **8** | **9** | **10** |  |
| --- | --- | --- | --- | --- | --- | --- | --- | --- | --- | --- | --- | --- |
| **No distress** | **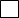** | **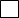** | **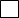** | **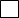** | **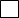** | **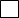** | **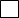** | **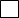** | **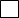** | **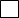** | **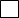** | **Extreme distress** |

**eHealth**

1. **How confident are you in using …?**
   1. **Digital media?**
      - Very insecure
      - Insecure
      - Neutral
      - Confident
      - Very confident
   2. **Platforms on the internet?**
      - Very insecure
      - Insecure
      - Neutral
      - Confident
      - Very confident
   3. **Digital devices (e.g. computers)?**
      - Very insecure
      - Insecure
      - Neutral
      - Confident
      - Very confident
   4. **Video conferencing systems (e.g. Zoom or Skype)?**
      - Very insecure
      - Insecure
      - Neutral
      - Confident
      - Very confident
2. **To what extent do each of the following statements apply to you …?**
   1. **I feel stressed by the constant availability via cellphone or e-mail.**
      - Does not apply
      - Rather does not apply
      - Partly/partly
      - Rather does apply
      - Does apply
   2. **I feel hassled by unwanted messages or e-mails.**
      - Does not apply
      - Rather does not apply
      - Partly/partly
      - Rather does apply
      - Does apply
   3. **I feel uncomfortable carrying a mobile device all the time.**
      - Does not apply
      - Rather does not apply
      - Partly/partly
      - Rather does apply
      - Does apply
   4. **I have concerns about using the Internet.**
      - Does not apply
      - Rather does not apply
      - Partly/partly
      - Rather does apply
      - Does apply
   5. **I fear that I could make an irrevocable mistake when using the Internet.**
      - Does not apply
      - Rather does not apply
      - Partly/partly
      - Rather does apply
      - Does apply
   6. **The Internet is something that worries me.**
      - Does not apply
      - Rather does not apply
      - Partly/partly
      - Rather does apply
      - Does apply
3. **What do you already know about Internet-based health promotion programs in cardiology …?**
4. **I can imagine what it is about.**
   - - Strongly disagree
     - Disagree
     - Neither
     - Agree
     - Strongly agree
5. **I know how such programs work.**
   - - Strongly disagree
     - Disagree
     - Neither
     - Agree
     - Strongly agree
6. **I know how to find such programs.**
   - - Strongly disagree
     - Disagree
     - Neither
     - Agree
     - Strongly agree
7. **Please rate the extent to which you agree or disagree with each statement.
   Explanation: The cardiology video consultation is the option of using video consultation with your specialist cardiologist**
   1. **I’d like to try the cardiological video consultation.**
      - Does not apply
      - Rather does not apply
      - Partly/partly
      - Rather does apply
   2. **I would use the cardiological video consultation if it was offered to me.**
      - Does not apply
      - Rather does not apply
      - Partly/partly
      - Rather does apply
      - Does apply
   3. **I would recommend the cardiological video consultation to friends of mine with a cardiological disease.**
      - Does not apply
      - Rather does not apply
      - Partly/partly
      - Rather does apply
      - Does apply
   4. **People close to me would approve of using the cardiological video consultation.**
      - Does not apply
      - Rather does not apply
      - Partly/partly
      - Rather does apply
      - Does apply
   5. **My general practitioner would approve the use of the cardiological video consultation.**
      - Does not apply
      - Rather does not apply
      - Partly/partly
      - Rather does apply
      - Does apply
   6. **My cardiological specialist would approve the use of the cardiological video consultation.**
      - Does not apply
      - Rather does not apply
      - Partly/partly
      - Rather does apply
      - Does apply
   7. **The cardiological video consultation could improve my general well-being.**
      - Does not apply
      - Rather does not apply
      - Partly/partly
      - Rather does apply
      - Does apply
   8. **Using the cardiological video consultation would allow me to save time (e.g. for traveling).**
      - Does not apply
      - Rather does not apply
      - Partly/partly
      - Rather does apply
      - Does apply
   9. **Using the cardiological video consultation could improve my health.**
      - Does not apply
      - Rather does not apply
      - Partly/partly
      - Rather does apply
      - Does apply
   10. **Using the cardiological video consultation would enable me to better integrate an appointment into my everyday life.**
       - Does not apply
       - Rather does not apply
       - Partly/partly
       - Rather does apply
       - Does apply
   11. **Using the cardiological video consultation would enable me to talk to the doctor more openly from a protected environment (e.g. from home).**
       - Does not apply
       - Rather does not apply
       - Partly/partly
       - Rather does apply
       - Does apply
   12. **Using the cardiological video consultation wouldn’t be an additional burden for me.**
       - Does not apply
       - Rather does not apply
       - Partly/partly
       - Rather does apply
       - Does apply
   13. **The cardiological video consultation would be easy for me to use and understand.**
       - Does not apply
       - Rather does not apply
       - Partly/partly
       - Rather does apply
       - Does apply
   14. **I could incorporate the use of the cardiological video consultation into my everyday life.**
       - Does not apply
       - Rather does not apply
       - Partly/partly
       - Rather does apply
       - Does apply
8. **Are you aware of the possibility of communicating with a doctor by video consultation?**

- Yes
- No

1. **Does your treating cardiologist already have the option of using video consultations?**

- Yes
- No
- I don’t know

1. **Have you already used the cardiology video consultation?**

- Yes, more than once
- Yes, once
- No

1. **If yes, for what kind of medical problems would you use the video consultation?**

(Multiple answers possible)

- Minor, unproblematic medical problems
- Questions about medication
- Questions about lifestyle changes
- Follow-up checks
- Issuing prescriptions or sick notes for work
- Discussion of findings/examination results
- Initial consultation

1. **How important would it be for you to know the doctor personally beforehand or to have established a personal relationship of trust when using the video consultation?**

- Very important
- Rather important
- Neutral
- Rather unimportant
- Not at all important

1. **What do you think are the main reasons for not using the cardiology video consultation?**

(Multiple answers possible)

- Prefer personal contact with the doctor
- Lack of technical requirements
- Fear of incorrect remote diagnoses
- Concern about lack of data protection

1. **In what form have you already used digital services in medicine?**(Multiple answers possible)

- Use of online pharmacies
- Use of online health portals/websites
- Mobile health apps (e.g. health tracker)
- Use of online support services
- Use of the electronic patient file
- Online doctor appointments
- Online ordering of medical prescriptions
- Telemedicine (e.g. video consultation, email contact)
- None

**eHealth Literacy**

1. **The following statements relate to health information on the Internet. Please read through the individual statements and mark how much you agree with each statement?**
2. **I know how to find websites with helpful health information.**
   - - Strongly disagree
     - Disagree
     - Neutral
     - Agree
     - Strongly agree
3. **I know how to use the internet to get answers to my health questions.**
   - - Strongly disagree
     - Disagree
     - Neutral
     - Agree
     - Strongly agree
4. **I know which websites with health information are available on the Internet.**
   - - Strongly disagree
     - Disagree
     - Neutral
     - Agree
     - Strongly agree
5. **I know where I can find helpful health information on the Internet.**
   - - Strongly disagree
     - Disagree
     - Neutral
     - Agree
     - Strongly agree
6. **I know how to use health information from the Internet in a way that helps me.**
   - - Strongly disagree
     - Disagree
     - Neutral
     - Agree
     - Strongly agree
7. **I am able to critically evaluate websites with health information.**
   - - Strongly disagree
     - Disagree
     - Neutral
     - Agree
     - Strongly agree
8. **I can distinguish between trustworthy and questionable websites with health information.**
   - - Strongly disagree
     - Disagree
     - Neutral
     - Agree
     - Strongly agree
9. **I feel confident using information from the internet to make decisions about my health.**
   - - Strongly disagree
     - Disagree
     - Neutral
     - Agree
     - Strongly agree

**Questionnaire: Original German-Version**

**Soziodemographie**

1. **Bitte geben Sie Ihr Alter an:**

_________ Jahre

1. **Bitte geben Sie Ihr Geschlecht an:**

| - Männlich | - Weiblich | - Divers |
| --- | --- | --- |

1. **Please state your current marital status**

- Verheiratet
- In Partnerschaft lebend
- Ledig
- Geschieden/Getrennt
- Verwitwet

1. **Was ist Ihr höchster Bildungsabschluss?**

- Schule beendet ohne Abschluss
- Volks-/Hauptschulabschluss
- Mittlere Reife
- Abitur bzw. Fachhochschule
- Hochschulabschluss
- Akademischer Grad
- Andere

1. **Bitte geben Sie Ihren aktuellen Beschäftigungsstatus an**

- Noch in der Ausbildung (z.B. Schule, Studium)
- Nicht erwerbstätig (z.B. arbeitssuchend, berufsunfähig)
- Krankgeschrieben
- Teilzeitbeschäftigt
- Voll erwerbstätig
- Berentet/ Pensioniert

1. **Sind Sie derzeit arbeitsunfähig?**

- Ja
- Nein

1. **Geben Sie bitte an, in welcher Art Stadt oder Gemeinde Sie wohnen:**

- Großstadt (ab 100 000 Einwohner)
- Mittelstadt (ab 20 000 Einwohner)
- Kleinstadt (ab 5 000 Einwohner)
- Landgemeinde (unter 5 000 Einwohner)

1. **Was beschreibt Ihre derzeitige Wohnsituation am besten?**

- Ich lebe allein
- Ich lebe mit meinem Partner/ Partnerin zusammen
- Ich lebe in einer Wohneinrichtung (Seniorenheim, betreutes Wohnen)

1. **Besitzen Sie einen Pflegegrad?**

- Nein, kein Pflegegrad
- Pflegegrad 1
- Pflegegrad 2
- Pflegegrad 3
- Pflegegrad 4
- Pflegegrad 5
- Weiß ich nicht

1. **Bitte geben Sie an, wie weit Sie ca. von Ihrem behandelnden Kardiologen entfernt wohnen.**

*Angabe in Kilometer. Wenn Sie über 100km entfernt wohnen, klicken Sie bitte 100 an. Z.B. Entfernung zu einer Praxis oder zu einem Krankenhaus (nutzen Sie den Schieberegler).*

**Kardiologische Anamnese**

1. **Besteht bei Ihnen eine Herzerkrankung (diagnostiziert durch einen Facharzt)?**

| - Ja | - Nein |
| --- | --- |

1. **Besteht bei Ihnen eine Erkrankung an anderen Gefäßen (diagnostiziert durch einen Facharzt)?**

| - Ja | - Nein |
| --- | --- |

1. **Besteht bei Ihnen die Diagnose einer Herzschwäche (diagnostiziert durch einen Facharzt)?**

| - Ja | - Nein |
| --- | --- |

1. **Hatten Sie mal einen Herzinfarkt?**

| - Ja | - Nein |
| --- | --- |

1. **Haben Sie bereits einen Stent oder Bypass am Herzen erhalten?**

| - Ja | - Nein |
| --- | --- |

1. **Leiden Sie an einer Herzrhythmusstörung?**

| - Ja | - Nein |
| --- | --- |

1. **Besitzen Sie einen Schrittmacher und/oder einen Defibrillator (kurz: Defi)?**

| - Ja | - Nein |
| --- | --- |

1. **Welche der folgenden Symptome sind bei Ihnen bekannt?**
   - Luftnot in Ruhe
   - Luftnot unter Belastung
   - Herzklopfen/ -stolpern
   - Wassereinlagerungen
   - Schwindel
   - Ohnmachtsanfälle
2. **9. Wie viele Etagen Treppensteigen oder Gehstrecke schaffen Sie ohne Pause, bevor Luftnot eintritt oder ein Beklemmungsgefühl in der Brust?**
   - 0
   - 1
   - 2
   - 3
   - 4
   - Keine Einschränkungen
3. **Wie viel Gehstrecke schaffen Sie ohne Pause, bevor Luftnot eintritt oder ein Beklemmungsgefühl in der Brust?**
   - 0 – 5 Minuten
   - 5 – 10 Minuten
   - 10 – 20 Minuten
   - 20 – 30 Minuten
   - 30 – 40 Minuten
   - > 40 Minuten
   - Keine Einschränkungen
4. **Rauchen Sie?**

- Ja
- Ich habe mal geraucht
- Nein

**Psychologische Anamnese**

1. **Liegt oder lag bei Ihnen eine psychische Erkrankung (diagnostiziert durch einen Arzt oder Psychotherapeuten) vor?**

- Ja
- Nein

1. **Wie oft fühlten Sie sich in den letzten 2 Wochen durch die folgenden Beschwerden beeinträchtigt?**
   1. **Wenig Interesse oder Freude an Ihren Tätigkeiten**
      - Nie
      - An einzelnen Tagen
      - An mehr als der Hälfte der Tage
      - Beinahe jeden Tag
   2. **Niedergeschlagenheit, Schwermut oder Hoffnungslosigkeit**
      - Nie
      - An einzelnen Tagen
      - An mehr als der Hälfte der Tage
      - Beinahe jeden Tag
   3. **Schwierigkeiten ein- oder durchzuschlafen oder vermehrter Schlaf**
      - Nie
      - An einzelnen Tagen
      - An mehr als der Hälfte der Tage
      - Beinahe jeden Tag
   4. **Müdigkeit oder Gefühl, keine Energie zu haben**
      - Nie
      - An einzelnen Tagen
      - An mehr als der Hälfte der Tage
      - Beinahe jeden Tag
   5. **Verminderter Appetit oder übermäßiges Bedürfnis zu essen**
      - Nie
      - An einzelnen Tagen
      - An mehr als der Hälfte der Tage
      - Beinahe jeden Tag
   6. **Schlechte Meinung von sich selbst; Gefühl ein Versager zu sein oder die Familie enttäuscht zu haben**
      - Nie
      - An einzelnen Tagen
      - An mehr als der Hälfte der Tage
      - Beinahe jeden Tag
   7. **Schwierigkeiten, sich auf etwas zu konzentrieren, z.B. beim Zeitungslesen oder Fernsehen**
      - Nie
      - An einzelnen Tagen
      - An mehr als der Hälfte der Tage
      - Beinahe jeden Tag
   8. **Waren Ihre Bewegungen oder Ihre Sprache so verlangsamt, dass es auch anderen auffallen würde? Oder waren Sie im Gegenteil "zappelig" oder ruhelos und hatten dadurch einen stärkeren Bewegungsdrang als sonst?**
      - Nie
      - An einzelnen Tagen
      - An mehr als der Hälfte der Tage
      - Beinahe jeden Tag
2. **Auf einer Skala von 0 bis 10, wie schätzen Sie Ihre körperliche Gesundheit (z. B. keine körperlichen Einschränkungen, Schmerzen) ein?**

|  | **0** | **1** | **2** | **3** | **4** | **5** | **6** | **7** | **8** | **9** | **10** |  |
| --- | --- | --- | --- | --- | --- | --- | --- | --- | --- | --- | --- | --- |
| **Sehr schlechte Gesundheiet** | **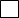** | **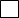** | **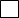** | **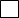** | **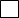** | **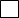** | **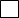** | **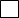** | **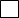** | **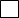** | **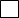** | **Sehr gute Gesundheit** |

1. **Auf einer Skala von 0 bis 10, wie schätzen Sie Ihre psychische Gesundheit (z. B. keine Angstgefühle, Niedergeschlagenheit) ein?**

|  | **0** | **1** | **2** | **3** | **4** | **5** | **6** | **7** | **8** | **9** | **10** |  |
| --- | --- | --- | --- | --- | --- | --- | --- | --- | --- | --- | --- | --- |
| **Sehr schlechte Gesundheiet** | **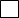** | **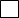** | **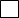** | **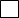** | **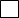** | **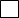** | **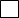** | **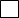** | **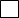** | **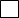** | **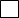** | **Sehr gute Gesundheit** |

1. **Auf einer Skala von 0 bis 10, wie schätzen Sie Ihre aktuelle Lebensqualität ein?**

|  | **0** | **1** | **2** | **3** | **4** | **5** | **6** | **7** | **8** | **9** | **10** |  |
| --- | --- | --- | --- | --- | --- | --- | --- | --- | --- | --- | --- | --- |
| **Sehr schlechte Lebensqualität** | **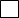** | **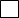** | **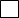** | **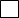** | **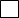** | **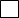** | **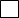** | **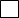** | **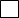** | **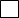** | **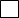** | **Sehr gute Lebensqualität** |

1. **Auf einer Skala von 0 bis 10, wie belastet fühlten Sie sich in der letzten Woche?**

|  | **0** | **1** | **2** | **3** | **4** | **5** | **6** | **7** | **8** | **9** | **10** |  |
| --- | --- | --- | --- | --- | --- | --- | --- | --- | --- | --- | --- | --- |
| **No distress** | **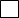** | **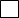** | **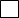** | **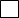** | **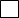** | **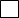** | **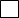** | **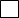** | **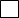** | **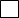** | **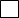** | **Extreme distress** |

**eHealth**

1. **Wie sicher sind Sie im Umgang mit...?**
   1. **Digitalen Medien?**
      - Sehr unsicher
      - Eher unsicher
      - Teils/teils
      - Eher sicher
      - Sehr sicher
   2. **Plattformen im Internet?**
      - Sehr unsicher
      - Eher unsicher
      - Teils/teils
      - Eher sicher
      - Sehr sicher
   3. **digitalen Endgeräten (z.B. Computer)?**
      - Sehr unsicher
      - Eher unsicher
      - Teils/teils
      - Eher sicher
      - Sehr sicher
   4. **Videokonferenzsystemen (z.B. Zoom oder Skype)?**
      - Sehr unsicher
      - Eher unsicher
      - Teils/teils
      - Eher sicher
      - Sehr sicher
2. **Inwieweit trifft jede der folgenden Aussagen auf Sie zu?**
   1. **Ich fühle mich durch die ständige Erreichbarkeit über das Handy oder per Mail belastet.**
      - Trifft nicht zu
      - Trifft eher nicht zu
      - Teils/Teils
      - Trifft eher zu
      - Trifft zu
   2. **Ich fühle mich von unerwünschten Nachrichten und E-Mails bedrängt.**
      - Trifft nicht zu
      - Trifft eher nicht zu
      - Teils/Teils
      - Trifft eher zu
      - Trifft zu
   3. **Ich fühle mich unwohl, dauerhaft ein mobiles Endgerät zu tragen.**
      - Trifft nicht zu
      - Trifft eher nicht zu
      - Teils/Teils
      - Trifft eher zu
      - Trifft zu
   4. **Ich habe Bedenken, das Internet zu benutzen.**
      - Trifft nicht zu
      - Trifft eher nicht zu
      - Teils/Teils
      - Trifft eher zu
      - Trifft zu
   5. **Ich fürchte, ich könnte einen unwiderruflichen Fehler bei der Nutzung des Internets machen.**
      - Trifft nicht zu
      - Trifft eher nicht zu
      - Teils/Teils
      - Trifft eher zu
      - Trifft zu
   6. **Das Internet ist etwas, das mich beunruhigt.**
      - Trifft nicht zu
      - Trifft eher nicht zu
      - Teils/Teils
      - Trifft eher zu
      - Trifft zu
3. **Was wissen Sie bereits über internetbasierte Programme zur Gesundheitsförderung in der Kardiologie?**
4. **Darunter kann ich mir schon etwas vorstellen.**
   - - Stimme gar nicht zu
     - Stimme eher nicht zu
     - Weder noch
     - Stimme eher zu
     - Stimme voll zu
5. **Ich weiß, wie solche Programme funktionieren.**
   - - Stimme gar nicht zu
     - Stimme eher nicht zu
     - Weder noch
     - Stimme eher zu
     - Stimme voll zu
6. **Ich weiß, wie ich solche Programme finden kann.**
   - - Stimme gar nicht zu
     - Stimme eher nicht zu
     - Weder noch
     - Stimme eher zu
     - Stimme voll zu
7. **Bitte bewerten Sie bei jeder Aussage, inwieweit Sie dieser zustimmen oder nicht zustimmen
   Erklärung: Die kardiologische Videosprechstunde ist die Möglichkeit der Nutzung von Videotelefonie mit Ihrer Fachärztin bzw. mit Ihrem Facharzt für Kardiologie**
   1. **Ich würde die kardiologische Videosprechstunde gerne ausprobieren.**
      - Trifft nicht zu
      - Trifft eher nicht zu
      - Teils/Teils
      - Trifft eher zu
      - Trifft zu
   2. **Ich würde die kardiologische Videosprechstunde nutzen, wenn sie mir angeboten würde.**
      - Trifft nicht zu
      - Trifft eher nicht zu
      - Teils/Teils
      - Trifft eher zu
      - Trifft zu
   3. **Ich würde die kardiologische Videosprechstunde Bekannten von mir mit einer kardiologischen Erkrankung empfehlen.**
      - Trifft nicht zu
      - Trifft eher nicht zu
      - Teils/Teils
      - Trifft eher zu
      - Trifft zu
   4. **Personen, die mir nahestehen, würden die Nutzung der kardiologischen Videosprechstunde gutheißen.**
      - Trifft nicht zu
      - Trifft eher nicht zu
      - Teils/Teils
      - Trifft eher zu
      - Trifft zu
   5. **Mein Hausarzt würde die Nutzung der kardiologischen Videosprechstunde gutheißen.**
      - Trifft nicht zu
      - Trifft eher nicht zu
      - Teils/Teils
      - Trifft eher zu
      - Trifft zu
   6. **Mein Facharzt für Kardiologie würde die Nutzung der kardiologischen Videosprechstunde gutheißen.**
      - Trifft nicht zu
      - Trifft eher nicht zu
      - Teils/Teils
      - Trifft eher zu
      - Trifft zu
   7. **Die kardiologische Videosprechstunde könnte mein allgemeines Wohlbefinden verbessern.**
      - Trifft nicht zu
      - Trifft eher nicht zu
      - Teils/Teils
      - Trifft eher zu
      - Trifft zu
   8. **Die Nutzung der kardiologischen Videosprechstunde würde es mir ermöglichen Zeit (z. B. für die Anreise) zu sparen).**
      - Trifft nicht zu
      - Trifft eher nicht zu
      - Teils/Teils
      - Trifft eher zu
      - Trifft zu
   9. **Die Nutzung der kardiologischen Videosprechstunde könnte meinen gesundheitlichen Zustand verbessern.**
      - Trifft nicht zu
      - Trifft eher nicht zu
      - Teils/Teils
      - Trifft eher zu
      - Trifft zu
   10. **Die Nutzung der kardiologischen Videosprechstunde würde es mir ermöglichen, einen Termin besser in meinen Alltag zu integrieren.**
       - Trifft nicht zu
       - Trifft eher nicht zu
       - Teils/Teils
       - Trifft eher zu
       - Trifft zu
   11. **Die Nutzung der kardiologischen Videosprechstunde würde es mir ermöglichen, mit dem Arzt offener aus einem geschützten Umfeld heraus (z. B. von Zuhause) zu sprechen.**
       - Trifft nicht zu
       - Trifft eher nicht zu
       - Teils/Teils
       - Trifft eher zu
       - Trifft zu
   12. **Die Nutzung der kardiologischen Videosprechstunde wäre keine zusätzliche Last für mich.**
       - Trifft nicht zu
       - Trifft eher nicht zu
       - Teils/Teils
       - Trifft eher zu
       - Trifft zu
   13. **Die kardiologische Videosprechstunde wäre für mich einfach zu bedienen und zu verstehen.**
       - Trifft nicht zu
       - Trifft eher nicht zu
       - Teils/Teils
       - Trifft eher zu
       - Trifft zu
   14. **Die Nutzung der kardiologischen Videosprechstunde könnte ich in meinen Alltag einbauen.**
       - Trifft nicht zu
       - Trifft eher nicht zu
       - Teils/Teils
       - Trifft eher zu
       - Trifft zu
8. **Sind Sie über die Möglichkeit einer Kommunikation mit einem Arzt mittels Videotelefonie informiert?**

- Ja
- Nein

1. **Besteht bei Ihrem behandelnden Kardiologen bereits die Möglichkeit zur Nutzung einer Videosprechstunde?**

- Ja
- Nein
- Weiß ich nicht

1. **Haben Sie die kardiologische Videosprechstunde bereits genutzt?**

- Ja, mehrfach
- Ja, einmal
- Nein

1. **Falls ja, für welche Art medizinischer Probleme würden Sie die Videosprechstunde nutzen?**

(Mehrfachnennung möglich)

- Kleinere unproblematische medizinische Probleme
- Fragen zur Medikation
- Fragen zu Lebensstiländerungen
- Verlaufskontrollen
- Dem Ausstellen von Rezepten oder Arbeitsunfähigkeitsbescheinigungen
- Besprechung von Befunden/ Untersuchungsergebnissen
- Erstgespräch

1. **Wie wichtig wäre es Ihnen bei der Nutzung der Videosprechstunde den Arzt zuvor bereits persönlich zu kennen bzw. ein persönliches Vertrauensverhältnis aufgebaut zu haben?**

- Sehr wichtig
- Eher wichtig
- Neutral
- Eher unwichtig
- Überhaupt nicht wichtig

1. **Was sind aus Ihrer Sicht die Hauptgründe die kardiologische Videosprechstunde nicht zu nutzen?**

(Mehrfachnennung möglich)

- Lieber persönlicher Kontakt zum Arzt
- Technische Voraussetzungen fehlen
- Angst vor fehlerhaften Ferndiagnosen
- Sorge vor fehlendem Datenschutz

1. **In welcher Form haben Sie bereits digitale Angebote in der Medizin genutzt?**(Mehrfachnennung möglich)

- Nutzung von Online-Apotheken
- Nutzung von Online-Gesundheitsportalen/ Websiten
- Mobile Health-Apps (z.B. Gesundheitstracker)
- Nutzung von Online-Unterstützungsangeboten
- Nutzung der elektronischen Patientenakte
- Online-Terminvereinbarung Arzt
- Online-Bestellung von ärztlichen Rezepten
- Telemedizin (z.B. Videosprechstunde, E-Mail-Kontakt)
- Keine

**eHealth Literacy**

1. **Die folgenden Aussagen betreffen Gesundheitsinformationen im Internet. Bitte lesen Sie die einzelnen Aussagen in Ruhe durch und kreuzen an, wie sehr Sie der jeweiligen Aussage zustimmen?**
2. **Ich weiß, wie ich Internetseiten mit hilfreichen Gesundheitsinformationen finden kann.**
   - - Stimme überhaupt nicht zu
     - Stimme eher nicht zu
     - Weder noch
     - Stimme eher zu
     - Stimme voll zu
3. **Ich weiß, wie ich das Internet nutzen kann, um Antworten auf meine Gesundheitsfragen zu erhalten.**
   - - Stimme überhaupt nicht zu
     - Stimme eher nicht zu
     - Weder noch
     - Stimme eher zu
     - Stimme voll zu
4. **Ich weiß, welche Seiten mit Gesundheitsinformationen im Internet verfügbar sind.**
   - - Stimme überhaupt nicht zu
     - Stimme eher nicht zu
     - Weder noch
     - Stimme eher zu
     - Stimme voll zu
5. **Ich weiß, wo ich im Internet hilfreiche Gesundheitsinformationen finden kann.**
   - - Stimme überhaupt nicht zu
     - Stimme eher nicht zu
     - Weder noch
     - Stimme eher zu
     - Stimme voll zu
6. **Ich weiß Gesundheitsinformationen aus dem Internet so zu nutzen, dass sie mir weiterhelfen.**
   - - Stimme überhaupt nicht zu
     - Stimme eher nicht zu
     - Weder noch
     - Stimme eher zu
     - Stimme voll zu
7. **Ich bin in der Lage, Internetseiten mit Gesundheitsinformationen kritisch zu bewerten.**
   - - Stimme überhaupt nicht zu
     - Stimme eher nicht zu
     - Weder noch
     - Stimme eher zu
     - Stimme voll zu
8. **Ich kann zwischen vertrauenswürdigen und fragwürdigen Internetseiten mit Gesundheitsinformationen unterscheiden.**
   - - Stimme überhaupt nicht zu
     - Stimme eher nicht zu
     - Weder noch
     - Stimme eher zu
     - Stimme voll zu
9. **Ich fühle mich sicher darin, Informationen aus dem Internet zu nutzen, um Entscheidungen in Bezug auf meine Gesundheit zu treffen.**
   - - Stimme überhaupt nicht zu
     - Stimme eher nicht zu
     - Weder noch
     - Stimme eher zu
     - Stimme voll zu
